# A randomized controlled double-blind clinical trial comparing versus placebo the effect of an edible algal extract (*Ulva Lactuca*) on the component of depression in healthy volunteers with anhedonia

**DOI:** 10.1186/s12888-018-1784-x

**Published:** 2018-06-28

**Authors:** François-André Allaert, Hervé Demais, Pi Nyvall Collén

**Affiliations:** 1 Chair of health claim evaluation EMMAS BSB & CEN Nutriment, 29 rue Sambin, 21000 Dijon, France; 2 Biovet Conseil, 1 Route du Linès, 56700 Merlevenez, France; 3 Amadéite SAS Pôle biotechnologique du Haut du Bois, 56580 Bréhan, France

**Keywords:** Depression, Anhedonia, Seaweed extract

## Abstract

**Background:**

The effects of the seaweed extract were evaluated on the animal model equivalent of depression compared with a control group treated with the carrier (spring water) and a reference group treated with Imipramine and showed significative effect. This clinical trial was intended to confirm in humans the potential efficacy identified in animals. The primary objective was to compare against a placebo the effect of Ulva L.L extract in healthy volunteers whose anhedonia was characterized by a component of depression.

**Methods:**

Single-centre double-blind randomized placebo-controlled clinical trial on parallel arms of two groups of 45 subjects. The study could include men or women aged 18 to 65 years with anhedonia characterized by a Snaith Hamilton Pleasure Scale score (SHAPS) of ≥5 and feeling low morale for at least four weeks characterized by a component of depression evaluated on the Quick Inventory of Depressive Symptomatology – Self Report (QIDS–SR). Evaluation criteria: QIDS-SR; Patient Global Improvement Impression (PGII) and Clinical Global Improvement Impression (CGII).

**Results:**

86 subjects were included in the trial: 42 in the placebo group and 44 Ulva group. At D84, QIDS-SR significantly decreased more in the Ulva.L.L. group than in the placebo group (p: 0.0389). This difference is essentially linked to an improvement of the sleep disorders (p: 0.0219), of the psychomotor consequences (p: 0.002) and of the nutrition behaviour (p: 0.0694). 90.1% have the feeling of being improved in the Ulva group vs 72.5% in the placebo group (p: 0.0114) and in parallel 90.9% of the practitioners have the feeling that the subject has improved vs 70.8% (p: 0.0214).

**Conclusion:**

This double-blind randomized placebo-controlled trial shows that daily intake for three months of a water-soluble extract of Ulva L.L. continues to significantly improve the component of depression of subjects presenting anhedonia compared with a placebo.

**Trial registration:**

Trial retrospectively registred on ClinicalTrial.gov under ID: NCT03545399 Date: 05/22/2018.

## Background

Anhedonia, defined as a loss of sensitivity when it comes to feeling pleasure, is observed in various personality disorders but also commonly and to a lesser extent in healthy subjects for whom it is a personality trait [1–7]. It may be attributable to a minor disturbance of the mesocorticolimbic reward system involving serotoininergic and dopaminergic mediators [8–10] that are expressed as ill-being by contrast with the term well-being and characterized notably by a decline in morale with a component of depression. Anhedonia is an isolated symptom and not a psychopathological state unlike depression of which it may a component but which requires the association of numerous other components to meet the DSM V (Diagnostic and statistical Manual of Mental Disorders) definition.

Faced with this symptom, psychotropic substances and especially antidepressants have been and still are used too often in particular because of the danger of addiction and dependence that continued intake may entail [3]. Faced with overconsumption, especially in France, and the need to reduce it (Government scheme for combatting drugs and addictive conduct 2013–2017. La documentation Française) many studies have been directed at research into nutriments or plant extracts that might have a similar action to antidepressants, acting on the same physiological mechanisms, but without inducing pharmacological dependence. They would supposedly provide a solution to the ill-being felt by people experiencing low morale with a component of depression but with no major depressive episode (MDE) as per the DSM V [11], which covers a very large proportion of people to whom antidepressants are still prescribed for want of a satisfactory scientifically-proven alternative.

The seaweed extract tested in this study and developed by OLMIX SA is part of this search for a non-medicinal alternative to combat low morale and its component of depression. Research has isolated a concentrated lyophilized and ground fraction of a water-soluble extract of an edible alga [12] named *Ulva lactuca* Linnaeus and more commonly known as sea lettuce. The extract obtained is rich in oligo-elements (up to 50%) and proteins (10–20%). A pre-clinical study has evaluated the antidepressant effect of the algal extract administered orally as a preventive treatment in three doses (10, 20 and 40 mg/kg/day) in Wistar adult male rats [13]. The effects of the seaweed extract were evaluated on the animal model equivalent of depression compared with a control group treated with the carrier (spring water) and a reference group treated with Imipramine in doses of 10 mg/kg/day. After 15 days of intake of the study products, the results show superior and comparable effects of Imipramine and seaweed extract at 20 and 40 mg/kg/day compared with the control. Moreover, a dose-dependent effect was observed in animals treated with the extract at doses of 10, 20 and 40 mg/kg/day [13]. To confirm in humans the potential efficacy of the product identified in animals, we conducted a randomized controlled double-blind clinical trial on the component of depression in healthy volunteers with anhedonia.

## Methods

### Study objectives

The primary objective of the trial was to compare against a placebo the effect of the product under test (Ulva L.L) in healthy volunteers whose anhedonia was characterized by a component of depression. The secondary objectives were to compare the repercussion of the anhedonia on work and activities, the investigating doctor’s opinion of the change in anhedonia, the subjects’ satisfaction with the treatment and tolerance of the product.

### Nature of the study

Single-centre double-blind randomized placebo-controlled clinical trial on parallel arms of two groups of 45 subjects. The Ethics committee approval by the French Ethics Committee CPP EST I has been obtained (approval registered under N° 2014/41 ID RCB: 2014-A01019–38) and the consent to participate for all subjects has been obtained by writing from the physician.

### Evaluation criteria

The presence and intensity of anhedonia were evaluated on the Snaith-Hamilton Pleasure Scale (SHAPS) [14, 15] and its component of depression by the Quick Inventory of Depressive Symptomatology-Self-Report (QIDS-SR) [16–19] and their repercussion on work and activities by item 7 of the Hamilton Depression Rating Scale (HAM-D) [20]. Subject satisfaction with the treatment was evaluated by the PGII (Patient Global Improvement Impression) and the investigating physician’s opinion by the Clinical Global Improvement Impression (CGII) [21].

### Course of the study

The trial comprised three visits: a medical enrollment visit on D0, an intermediate visit on D28 and a final visit on D84. A psychologist made a telephone call after 7 days to check there was no sudden change in the subject’s depressive state. During the visits, the subject was examined by a doctor and the psychometric tests were administered by a psychologist. Outside the visits, the subject was monitored by a self-reporting questionnaire in the manner described below.

After obtaining informed written consent and validating the inclusion and exclusion criteria, notably the absence of MDE as per DSM V, the investigator enrolled the subject in the trial. The physician described the subject’s demographic and general clinical characteristics, the main medical and surgical history and any current treatment if those pathologies were still present, and any previous antidepressant, anxiolytic or neuroleptic treatments. The doctor asked the subject to complete the SHAPS and QIDS –SR questionnaires and item 7 of the HAM-D. The doctor then handed the subject self-evaluation questionnaires explaining how to complete them and the dates at which they were to be completed. The physician gave the patient a batch of the placebo or a batch of *Ulva lactuca* as per the randomization. The doctor told the subject a pyschologist would be in touch after 1 week by telephone to check no DSM V severity criterion had arisen.

The investigating doctor saw the subject again at the four-week intermediate vist and at the final visit at the end of the 12th week of follow-up. The doctor collected the self-questionnaires at these visits and the same items as at the enrollment visit were recorded by the physician for describing how the items had evolved and notably the SHAP scale score, the QIDS–SR and item 7 of the HAM-D. In addition, the physician recorded the subject’s satisfaction with the treatment evaluated on the PGII (Patient Global Improvement Impression), reported any undesirable affects and gave his/her own opinion on the efficacy of treatment using the Clinical Global Improvement Impression (CGII).

### Selection of the study population

#### Inclusion criteria

The study could include men or women aged 18 to 65 years with anhedonia characterized by a SHAPS scale score of ≥5 and feeling low morale for at least 4 weeks characterized by a component of depression evaluated on the QIDS–SR.

#### Exclusion criteria

The study could not include subjects with a major depressive episode as per the DSM V, with a HAM D > 8, already on anxiolytic, antidepressor, neuroleptic or any other medicinal treatment liable to influence mood (e.g. benzodiazepines) in the last 30 days and those who consumed illegal psychotropic substances, with daily alcohol intake of more than three glasses of wine, or having taken in the last 15 days products liable or presented as liable to act on stress, anxiety, mood or mental well-being. Also excluded were people with post-traumatic stress disorder (particularly stressful event, death, death threat, serious injuries, etc.), presenting with unstable or severe comorbidity or psychiatric disorder. In addition, the product under study being a seaweed extract, people with an allergy or intolerance to seafood were excluded.

### Treatment

#### Products under study

The test dose of seaweed extract was 6.45 mg per kg body weight. The selected daily dose was three capsules per day dosed at 130 mg for subjects weighing 50 to 75 kg, four capsules per day for subjects weighing 70 to 90 kg and five capsules per day for subjects weighing 90 to 110 kg. The product was taken in a single daily dose with the evening meal to be swallowed with a glass of water. It is extracted from *Ulva lactuca* by grinding and pressing, pasteurization and ultracentrifugation on ceramic membrane before being concentrated and drying. Then maltodextrine is added and the capsules are filled up with the resulting mixture. Placebo capsules are stricly identical and filled with maltodextrine only.

#### Prohibited treatments

Any product corresponding to the exclusion criteria was prohibited during the study: food supplements, functional foods, phytotherapy products and any other products liable or presented as liable to act on stress, anxiety or mental well-being other than the study product; anxiolytic, antidepressant, neuroleptic treatment or any medicinal treatment liable to influence mood and anhedonia (e.g. benzodiazepines) during the study. All other treatments than those prohibited were authorized and had to be recorded.

### Statistical analyses

#### Definition of populations

All of the minor and major deviations from the protocol were used to identify the different populations. The efficacy analysis pertains in turn to the intention to treat (ITT) and the per protocol (PP) population. The ITT study was performed on all subject having taken the product at least once and for the primary criterion could be evaluated at one post-enrollment evaluation visit at least (modified ITT). Where evaluation parameter data were missing, the analysis included the last available evaluation by the “Last Observation Carried Forward (LOCF)” technique. The PP evaluation was conducted on volunteers included in the ITT who took the product up to D84 and were seen again at D84, excluding subjects who violated the protocol in any major way. The tolerance analysis pertains to the population including all subjects who took the product under study at least once.

#### Statistical methods

The quantitative data were described by numbers, means, standard deviations, medians, minima and maxima. The qualitative data were described by their absolute and relative frequency. Means between groups at enrollment were compared using analysis of variance (or by the Kruskal Wallis test if the distributions were not normal distributions). Percentages were compared by the chi-square test (or Fisher’s exact test if the conditions for the chi-square test were not satisfied). The quantitative evaluation criteria were compared between the groups at different times by analyses of variance on paired series with two time and treatment factors with interactions between D0 and D84. The evaluation criteria were compared between groups by the chi-square test (or Fisher’s exact test if the conditions for applying the chi-square test were not met). Undesirable effects observed were described by their frequency and nature, which were compared by a chi-square test or Fisher’s exact test if the conditions for the chi-square test were not met).

#### Determination of sample size

The computation of sample numbers depended on identifying a 1.4-point difference in scores on the SHAPS scale between the placebo and the Ulva L.L. groups after 12 weeks. According to the calculation made with Nquery software, with a risk alpha = 0.05, power of 80% and a standard deviation of 2,5 [22], the number of subjects to be enrolled was 40 per arm, making 90 subjects in total to allow for lost sight subjects.

## Results

### Distribution of subjects

**Fig. 1:**
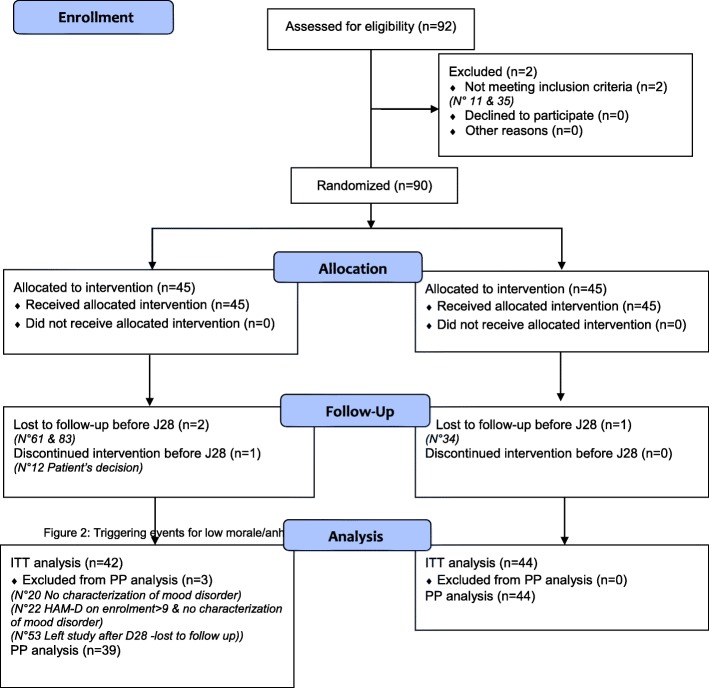
Distribution of subjects in the study

A total of 90 subjects were recruited: 45 in the placebo group and 45 in the Ulva L.L. group on enrollment (D0). Three subjects in the placebo group and one subject in the Ulva L.L. group left the study before D28 and could not be evaluated. The ITT analysis relates to 42 subjects in the placebo group and 44 in the Ulva L.L. group. Three subjects present a major deviation in the placebo group, as indicated in Fig. 1, and none in the Ulva L.L. group. The PP analysis relates to 39 subjects in the placebo group versus 44 in the Ulva L.L. group. The tolerance population relates to the 90 subjects who took at least one dose of the study products, 45 in each group (Fig. 1).

### Description of subjects at enrollment (*n* = 86)

The 86 subjects included in the ITT study were aged 40.2 ± 13.6 years and nearly two-thirds of them were women (65.1%). Their BMI was 25.2 ± 4.3 kg/m^2^ and 38.4% were overweight and 11.6% were obese. They had 1.2 ± 1.2 children on average and three-quarters of them (75.6%) lived in urban settings. Most (51.1%) were living alone, being either single (39.5%) or divorced (11.6%). Their level of education was below Bachelor’s degree (baccalauréat) for 29.1%, from Bachelor’s degree to 2 years in higher education for 39.5% and more than 2 years in higher education for 31.4%. Two-thirds of them (67.6%) had an occupation the nature of which was similar to that of the national population. In terms of lifestyle, 16.2% of subjects had been (8.1%) or still were (8.1%) smokers, 10.5% drank alcohol (9.3% 1 to 3 glasses and 1.2% more than 3 glasses) and 80.8% drank coffee or tea (67.4% from one to three cups and 12.8% more than three cups). It was also recorded that 54.7% engaged in a physical activity (47.7% 1 to 3 times per week and 7.0% more than 3 times per week).

**Fig. 2:**
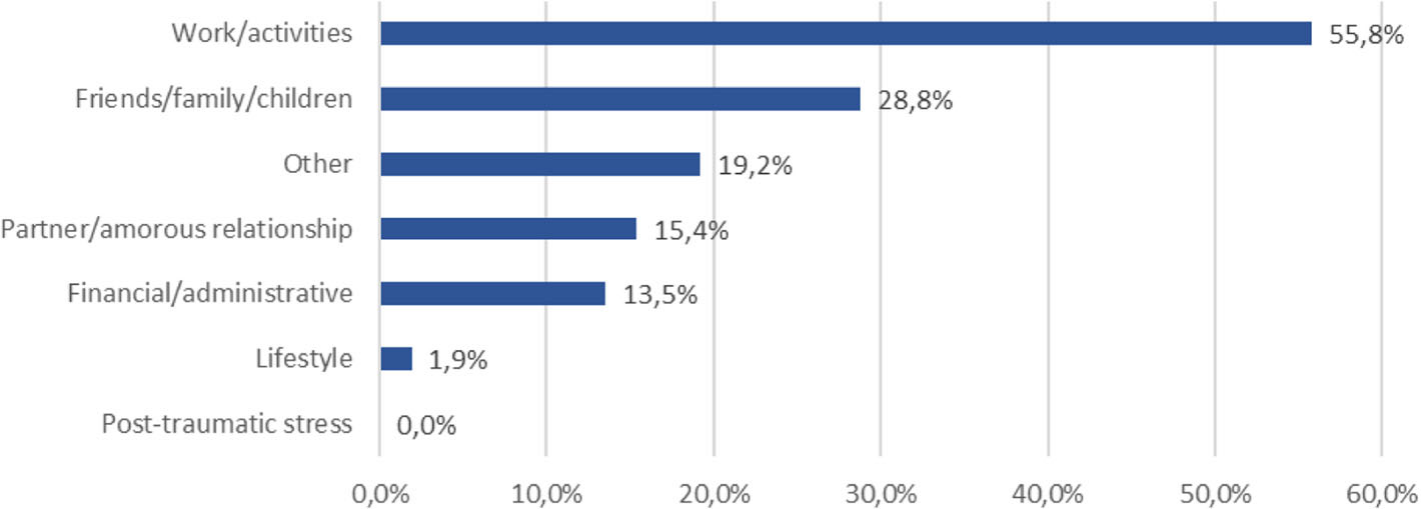
Triggering events for low morale/anhedonia

Anhedonia was felt by subjects for 19.9 ± 16.4 weeks on average and was anticipatory anhedonia for 91.8% of subjects and/or consummatory anhedonia for 70.6%. Low morale was also felt for 18.9 ± 14.5 weeks. The low morale was characterized by an unusual aversion to everyday chores for 90.7% of subjects and unusual resignation due to the difficulties of everday life for 30.2%. A triggering event was behind the low morale and anhedonia in nearly two-thirds of subjects (60.5%) essentially in conjunction with problems relating to work (55.8%), children (28.8%), affective relations (15.4%), time of year (15.4%) and financial difficulties (13.5%) (Fig. 2); the same subject could report several sources. 18.6% of the subjects had already experienced on average 1.2 ± 0.5 earlier depressive episodes lasting on average 35.3 ± 38.2 weeks. Subjects’ sociodemographic characteristics and anhedonic histories were statistically comparable in the two groups as were their clinical characteristics upon enrollment.

On the Snaith-Hamilton pleasure scale (SHAPS), almost all subjects presented severe anhedonia characterized by a score of more than 5 (92.9% in the placebo group vs 90.9% in the Ulva L.L group and with comparable mean values of 9.0 ± 2.4 vs 9.0 ± 2.4, respectively. The QIDS-SR inventory score of depressive symptoms was 11.2 ± 4.3 in the placebo group vs 12.9 ± 3.9 in the Ulva L.L. group (p: 0.0708) out of a maximum score of 27, reflecting signs of slight to moderate depression. The presence of a component of depression was confirmed by the results of the HAMD Hamilton rating scale for depression with mean values of 6.2 ± 2.0 vs 6.2 ± 1.9 respectively; p: 0.7743.

### Comparison of the change in subjects’ psychological state

Anhedonia improved significantly (*p* < 0.0001) in both the placebo and Ulva L.L. groups by 4.3 ± 3 points between D0 and D28. This improvement was still ongoing at D84 but more so in the Ulva L.L. group than in the placebo group without, however, being statistically significant: 6.5 ± 2.8 vs 5.6 ± 3.6 (p: 0.1921). This greater improvement in the Ulva L.L. group was also reflected in the frequency of severe anhedonia that persisted in 24.4% of the placebo group subjects versus 13.6% of the Ulva L.L. group subjects but again without being statistically significant (p: 0.2050).

**Fig. 3:**
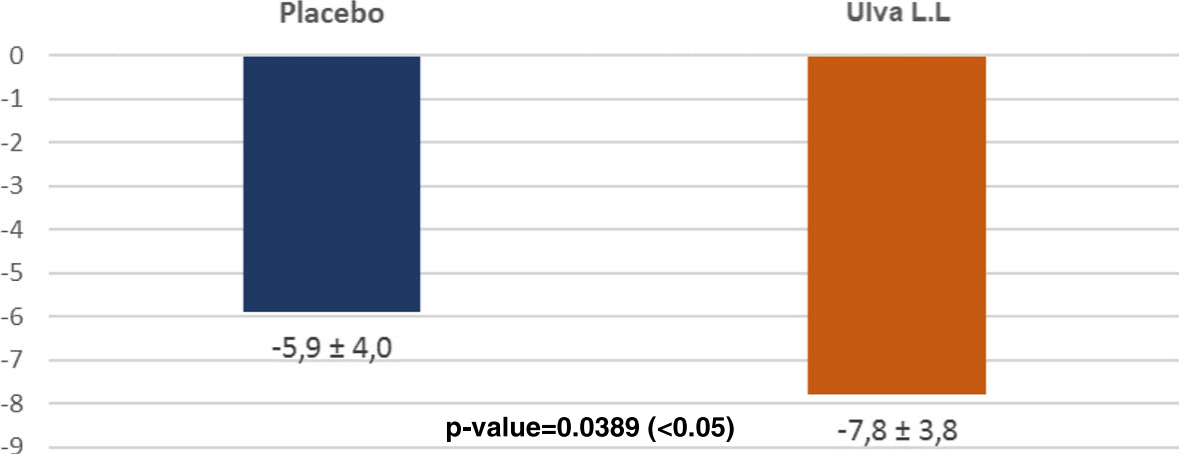
Change in total QIDS-SR score between D0 and D84 for each treatment

Results for depressive symptoms were, by contrast, far more significant. Analysis of the total QIDS-SR depressive symptoms inventory score showed significant improvements in the placebo and Ulva L.L. groups as from D7 in both goups. These improvements were comparable at D7 (3.5 ± 3.8 vs 3.6 ± 3.7; p: 0.9072) and D28 (4.5 ± 3.7 vs 4.9 ± 3.3; p: 0.6112) but became significantly greater in the Ulva L.L. group than the placebo group by D84 (7.8 ± 3.8 vs 5.9 ± 4; p: 0.0389) (Table 1; Fig. 3).

**Table 1:** Comparison of changes in QIDS-SR

Product	D0 m ± SD IC95%:	D7 m ± SD IC95%:	Delta D0/D7 m ± SD IC95%:	D28 m ± SD IC95%:	Delta D0/28 m ± SD IC95%:	D84 m ± SD IC95%:	Delta D0/84 m ± SD IC95%:	
Placebo (*n* = 38)	11.2 ± 4.3 [9.8; 12.6]	7.8 ± 3.7 [6.5; 9]	3.5 ± 3.8 [2.2; 4.7]	6.7 ± 4.2 [5.3; 8.1]	4.5 ± 3.7 [4.1; 6.5]	5.3 ± 3.7 [4.1; 6.5]	5.9 ± 4.0 [4.6; 7.2]	
Ulva L.L (*n* = 42)	12.9 ± 3.9 [11.7; 14.1]	9.3 ± 4 [8.1; 10.6]	3.6 ± 3.7 [2.4; 4.7]	8.0 ± 3.6 [6.8; 9.1]	4.9 ± 3.3 [3.9; 6]	5.1 ± 2.8 [4.3; 6]	7.8 ± 3.8 [6.6; 9.0]	
Anova	*p*-value: 0.0708	*p*-value: 0.0748	*p*-value: 0.9072	*p*-value: 0.1513	*p*-value: 0.6112	*p*-value: 0.8118	*p*-value: 0.0389

This greater improvement in the total QIDS-SR depressive symptoms inventory score was accounted for in particular by greater changes in the Ulva L.L. group than in the placebo group for three of its symptomatic dimensions: sleep disturbance, psychomotor change and changes in appetite or weight.

**Fig. 4:**
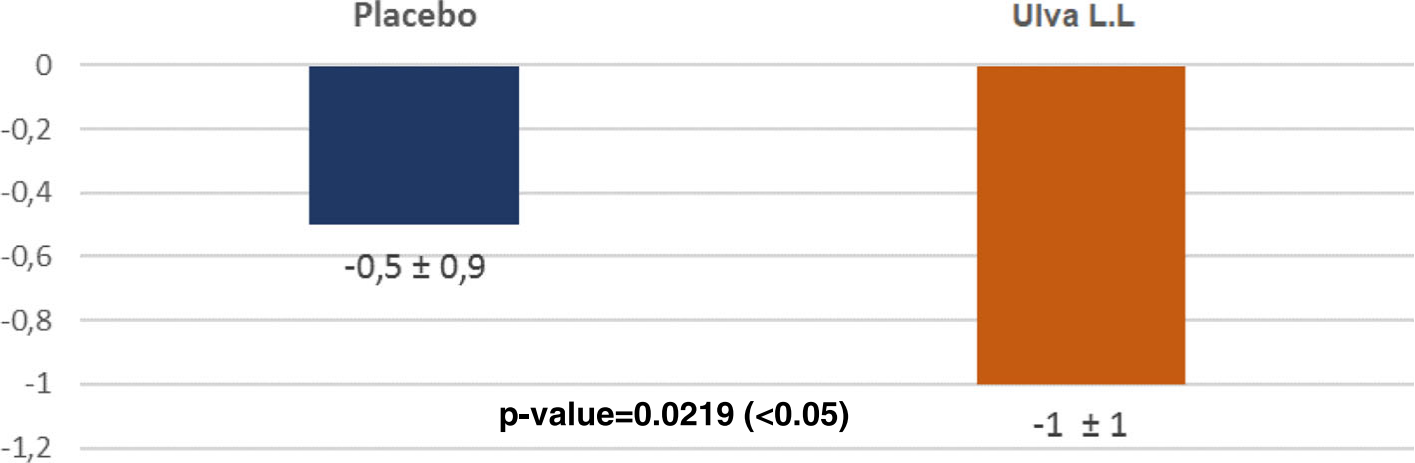
Changes in the QIDS-SR INSOMNIA/HYPERSOMNIA domain between D0 and D84 for each treatment

QIDS-SR “Sleep disturbance” improved significantly in the placebo and Ulva L.L. groups but comparably so between the groups at D7 (0.3 ± 0.6 vs 0.5 ± 0.9; p: 0.1623) and D28 (0.5 ± 1 vs 0.5 ± 0.9; p: 0.1623). However, it remained stable in the placebo group at D84 whereas it continued to improve in the Ulva L.L. group, with the difference between groups reaching the statistically significant level of 0.5 ± 0.9 vs 1.0 ± 1.0; p: 0.0219) (Fig. 4).

**Fig. 5:**
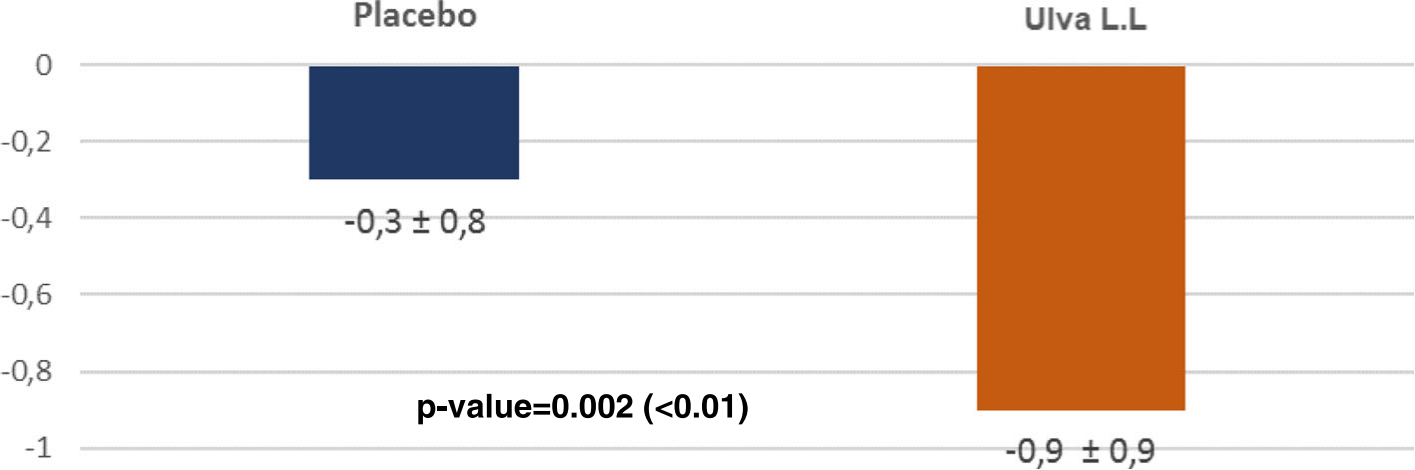
Changes in the QIDS/SR PSYCHOMOTOR RESTLESS/ SLOW DOWN domain between D0 and D84 for each treatment

“Psychomotor changes” improved significantly in both groups but whereas the changes were comparable at D7 (0.1 ± 0.7 vs 0.3 ± 0.9; p: 0.1684), they diverged significantly by D28. Psychomotor changes remained stable in the placebo group in this interval but continued to improve in the Ulva L.L. group becoming statistically significant (0.1 ± 0.8 vs 0.6 ± 0.9; p: 0.018) and the gap continued to widen at D84 (0.3 ± 0.8 vs 0.9 ± 0.9; p: 0.002) (Fig. 5).

**Fig. 6:**
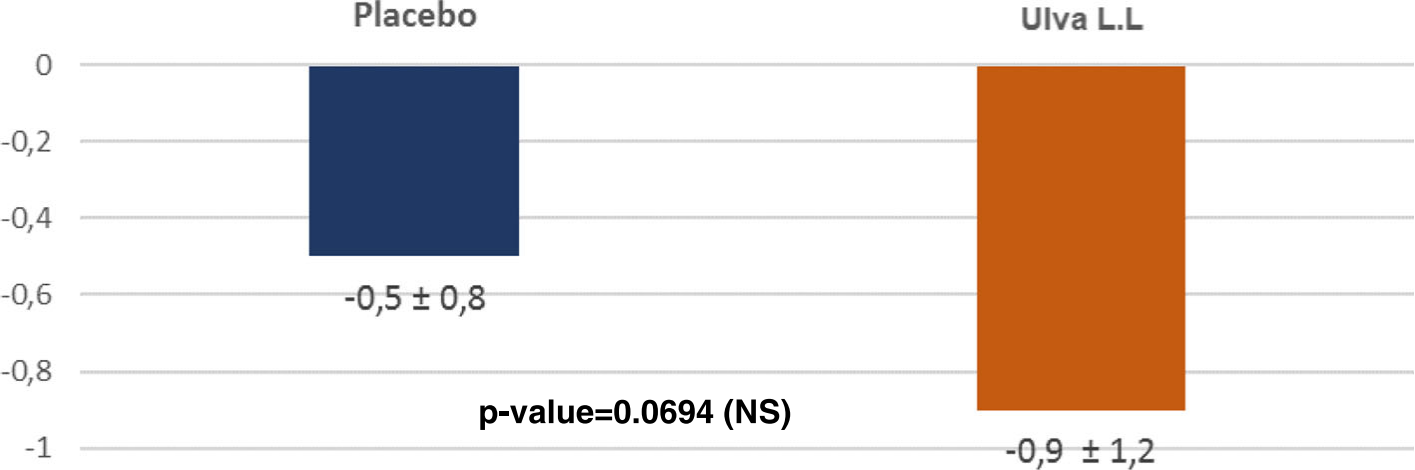
Changes in the QIDS-SR CHANGE IN APPETITE AND WEIGHT domain between D0 and D84 for each treatment

Likewise, whereas the change in appetite and weight improved significantly in the placebo and Ulva L.L. groups comparably at D7 (0.5 ± 1 vs 0.7 ± 1.2; p: 0.3811) and D28 (0.5 ± 1 vs 0.8 ± 1.2; p: 0.1801) this improvement continued in the Ulva L.L. tending to reach the statistically significant level 0.5 ± 0.8 vs 0.9 ± 1.2; p: 0.0694 whereas it remained stable in the placebo group (Fig. 6).

The other symptomatic components of the QUIDS-SR improved significantly but comparably for the two groups whether for mood, concentration and decision-making, self-outlook, involvement in activities or fatigability. Suicidal ideation was too scarcely present in this population of subjects with anhedonia and low morale for their change to be interpreted.

Item 7 of the HAM-D score centred on work improved significantly (*p* < 0.01) in both the placebo and Ulva L.L. groups by 0.4 ± 0.8 points and 0.6 ± 0.9 respectively between D0 and D28 and by 0.7 ± 1 and 0.9 ± 1.1 points by D84 but the higher values recorded in the Ulva L.L. group were not statistically significant.

**Fig. 7:**
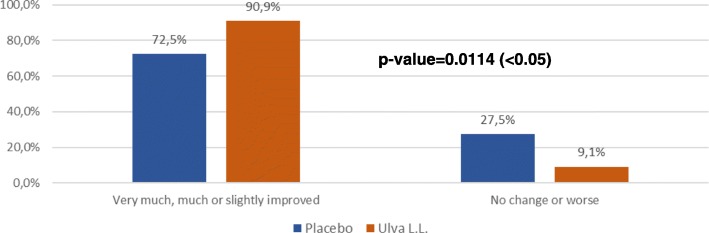
PGII at D84 for each treatment

The percentage of subjects feeling improvement at D28 was higher in the Ulva L.L. group than in the placebo group (65.9% vs 59.5%) which was close to being statistically significant (p: 0.0651) and the difference continued to increase becoming statistically significant by D84 (90.9% vs 72.5%;p: 0.0114) (Fig. 7).

**Fig. 8:**
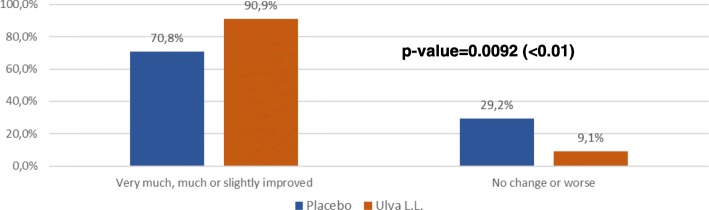
CGII at D84 for each treatment

The percentage of subjects that clinicians judged to have improved was comparable in the Ulva L.L. and placebo groups (81.8% vs 75.6%; p: 0.4987) at D28 but the percentage rose signifiantly at D84 in the Ulva L.L. group (90.9% vs 70.7%; p: 0.0092) (Fig. 8).

### Evaluation and comparison of tolerance and compliance

Twenty-eight undesirable events occurred in the course of the study concerning a total of 23 volunteers: 9 (20%) in the placebo group and 14 (14.9%) in the Ulva L.L. group (31.1%) (p: 0.2269). These events are essentially respiratory, thoracic and mediastinal affections (42.9%), gastro-intestinal affections (32.1%) and nervous system affections (10.7%) in comparable proportions in both groups. They were slight in 39.3% of cases, moderate in 39.3%, and severe in 21.4% and again in comparable proportions in both groups. Among the undesirable events, it was judged that only three of them for each group could not be excluded. In the placebo group an instance of diarrhoea, increased appetite and abdominal distensions were each deemed to be possibly related to the treatment. In the Ulva L.L. group, an instance of gastroentiritis, increased appetite and abdominal distensions were judged to be possibly treatment-related. No serious undesirable event was reported. The compliance was measured by the number of capsules people brought back at the last visit and was good with 96.5% of the subjects having an intake of more than 80% without difference between groups.

## Discussion

The major finding of this double-blind randomized control trial is to show the positive effect of the seaweed extract on the component of depression of anhedonic patients measured by the QIDS-SR benchmark despite a substantial placebo effect in the control group. By D84, the total QIDS-SR score. Significantly decreased more in the Ulva.L.L. group than in the placebo group (p: 0.0389). This difference is essentially linked to an improvement of the sleep disorders (p: 0.0219), of the psychomotor consequences (p: 0.002) and of the nutrition behaviour (p: 0.0694).. Some 90.1% of subjects said they felt an improvement with Ulva L.L. versus 72.5% in the placebo group (p: 0.0114) and similarly 90.9% of doctors judged the subjects to have improved versus 70.8% (p: 0.021). It should be pointed out that this effect shows up despite a substantial placebo effect that continued practically until D28 and then declined whereas the depressive symptoms continued to improve in subjects taking the water-soluble extract of Ulva L.L. This gradual improvement perceived by subjects and physicians is consistent with the gradual effect observed in the change in the QIDS-SR total depressive symptom inventory score and three of its subsections. The results attest to the effect of a dietary supplement that requires a relatively long intake period to produce a gradual physiologically significant change and of a placebo whose effect gradually declines with time. The size of the effect is also worth pointing out. In a phase II a trial on the efficacy of a combination of dextromethorphane and quinidine (Q), the observed improvement was − 5.9 ± 6.6 [23] and in a trial comparing Quetiapine against a placebo the difference in the QIDS-SR effect was 3.7 [24].

This effect on the component of depression of anhedonia thus seems to be clearly demonstrated and corroborates the results of the preclinical study evaluating the antidepressant effect of the seaweed extracts administered orally in adult male Wistar rats [13]. That study compared the seaweed extract at doses of 10, 20 and 40 mg/kg/day on groups of 12 rats against a control batch treated with the carrier (springwater) and a reference batch treated with Imipramine at doses of 10 mg/kg/day. The primary objective was to determine the efficacy of the extract on depression after 15 days of treatment on the principle of the Behavioural Despair Test (BDT) that is the benchmark for screening substances with an antidepressent effect. The results showed animals treated with Imiparmine and seaweed extract were less resigned than those treated with the placebo. A dose-dependent effect was observed for the seaweed extract.

Tolerance of the seaweed extracts was good and comparable with that of the placebo. This supports two toxicity studies conducted before the clinical trial: evaluation of acute toxicity and evaluation of sub-chronic toxicity by ingestion [25, 26]. Acute toxicity of the seaweed extract taken orally was evaluated with a single limit dose of 2000 mg/kg in 12 male and female Wistar rats monitored for 15 days. Two experimental series of six rats were studied. No mortality was observed and no abnormal behaviour was observed in the two-experimental series for male and female rats before and 1, 2, 3, 4, 5 and 6 h after administration of the single oral dose until completion of the experiment. No direct or induced significant weight loss was observed [25]. No anomaly of any organ was observed at autopsy. Subchronic toxicity of the seaweed extract was evaluated by oral administration at doses of 250, 500 and 1000 mg/kg/day for 28 days in male and female Wistar rats. No mortality, no abnormal behaviour, no body weight loss, no change in body weight change, no difference in food and water consumption, no opthalmological anomaly, no difference in functional tests and no histopathological anomaly was observed after 28 days of treatment. This good tolerance is an important factor for this product that could be an alternative to benzodiazepines for light to moderate depressive symptoms or at least as first-line treatment so they could be reserved for more severe situations. Further clinical studies must be conducted to confirm this since the registration of products whether as dietary supplements (health claims) or medication (marketing authorization) require at least two convergent clinical trials.

A review of the litterature does not retrieve some similar studies using alga extract. However, it is noticeable that the research of alternative natural products to the benzodiazepines is a growing research way which corresponds to a real patients’ and societal need and that clearly appear in the increasing number of published papers presenting good quality randomised clinical trials on that subject in the reference data bases like Pubmed. Among these research, can be mentioned the works of Mosaffa-Jahromi M [27] on the effectiveness of anise oil for Treatment of Mild to Moderate Depression in Patients With Irritable Bowel Syndrome, of Pasalar M [28] on the efficacy of jollab in the treatment of depression in dyspeptic patients, of Anushiravani M [29] comparing the effectiveness of a combined herbal drug based on Echium amoenum with Citalopram in the treatment of Major Depressive Disorder or those of Lopresti AL [30] on a standardised extract from saffron (*Crocus sativus* L.) for the treatment of youth anxiety and depressive symptoms. Unfortunately all these works do not use the same criteria that we used to evaluate the effect on depression and do not target the same profile of depressive patients which do not allow real efficacy comparison.

The status of the product is another point of discussion. As this pivotal study has demonstrated the action of Ulva L.L. extracts on depressive symptoms in humans, it now remains to be seen whether to continue with it as a dietary supplement with the difficulty of this positioning for depression or to shift towards a plant-based medicinal status for which the product has potential. Identifying the substance contained in the seawaeed extract and the source of the effect would be an added benefit whether for the dietary supplement case or the plant-based medication case, but is not a mandatory requirement.

## Conclusion

This double-blind randomized placebo-controlled trial shows that daily intake for 3 months of a water-soluble extract of Ulva L.L. continues to significantly improve the component of depression of subjects presenting anhedonia compared with a placebo. This opens up perspectives for its potential use in everyday clinical care particularly as it would avoid the undesirable effects of medicinal drugs currently used. It is worth underscoring that this effect is shown despite a substantial placebo effect that persisted up to D28 and then diminished whereas the depressive symptoms continued to improve in subjects taking the water-soluble Ulva L.L. extract.

## Abbreviations

BDT: Behavioural Despair Test
BMI: Body mass index
CGII: Clinical Global Improvement Impression
CGII: The Clinical Global Improvement Impression
DSM V: Diagnostic and statistical manual of mental disorders) – 5th edition
HAM-D: Hamilton rating scale for depression
ITT: Intention to treat
LOCF: Last Observation Carried Forward
MDE: Major depressive episode
PGII: Patient Global Improvement Impression
PGII: Patient Global Improvement Impression
PP: Per protocol
QIDS-SR: Quick Inventory of Depressive Symptomatology -Self Report
SHAPS: Snaith Hamilton Pleasure Scale
